# Factors Influencing the Oral Health Behaviours of Autistic Children and Young People: A Qualitative Study

**DOI:** 10.1111/hex.70130

**Published:** 2024-12-17

**Authors:** Jo Erwin, Sarah Neill, Tara Vassallo, Isaac Vassallo, Rob Witton, Martha Paisi

**Affiliations:** ^1^ Peninsula Dental School University of Plymouth Plymouth UK; ^2^ School of Nursing and Midwifery University of Plymouth Plymouth UK; ^3^ Plymouth Institute of Education University of Plymouth Plymouth UK; ^4^ National Autistic Society – Plymouth & District Branch Plymouth UK; ^5^ School of Engineering, Computing and Mathematics University of Plymouth Plymouth UK; ^6^ Peninsula Dental Social Enterprise Plymouth UK

## Abstract

**Background:**

Poor oral health (OH) in childhood can cause pain, affect quality of life and significantly impact adult OH. Autistic children and young people (CYP) experience inequalities in OH and are at higher risk of cavities and gum disease than neurotypical CYP.

**Objective:**

To provide evidence and insights into the factors affecting the OH behaviours of ACYP from the perspective of ACYP, their parents/carers and Dental Health Professionals (DHPs).

**Method:**

Semi‐structured online or face‐to‐face interviews were conducted with CYP, parents/carers and DHPs in Southwest England.

**Results:**

Nineteen ACYP (aged 5–18 years old), 20 parents/carers and 16 DHPs were interviewed. The themes identified were as follows: Sensory sensitivities and diet; Toothbrushing ‘battles’; Coping with the stresses of daily life in a neurotypical world; Awareness of autism and understanding of challenges to good OH; The impact on parents of trying to care their child's OH; CYP and parent/carers difficulties in obtaining OH support; What ‘good support’ looks like.

**Conclusions:**

An increased awareness among DHPs, health and social care professionals of autism and the challenges to OH that CYP face is needed. Providing early support, strategies and resources to CYP and their parents/carers has the potential to help establish positive OH behaviours.

**Public Contribution:**

The research team worked closely with members of the autistic community on this project. The methods used in this project were developed together with a research advisory group which included four ACYP (aged 9–17) and their parents. The team also worked with members of a youth club for ACYP aged 10–17 years old. All research documents and materials were co‐produced. Members of the advisory group (T.V. and I.V.) contributed to the interpretation of the research findings and the writing of this manuscript.

## 1 Introduction

Autistic children and young people (ACYP) and adults experience significant health inequalities [1, 2] including inequalities in oral health (OH) [3]. Poor OH in childhood can cause pain, discomfort and social embarrassment and can affect OH into adulthood [4]. Behavioural and social factors that impact OH include diet, oral hygiene practices such as toothbrushing, OH knowledge, access to dental care, income and levels of deprivation [5, 6, 7].

Studies show ACYPs have high levels of unmet dental treatment needs [3] and are more likely than neurotypical CYP to receive treatment under general anaesthesia [8]. ACYP have a higher risk of dental caries, gum disease and poor oral hygiene levels compared to their neurotypical peers [9]. A systematic review of the literature of the factors influencing the OH behaviours of ACYP highlighted sensory sensitivities, difficulties with change of routine and non‐autism‐related cognitive and motor skills differences as important factors affecting the maintenance of oral hygiene [10]. Diet and the use of tricyclic medications can also contribute to poor OH [11, 12].

The reduction of OH inequalities for CYP is an international and national priority and there has been increasing focus on ACYP and those with learning disabilities [13, 14]. Core20PLUS5 is a national NHS England approach to support the reduction of health inequalities. It targets OH service improvements for children under ten years of age, including autistic children [15].

Research is needed to identify how and why ACYP experience OH inequalities. A systematic review by the authors [10], showed how rare it is to include the voice of ACYP in qualitative studies of OH with only one study [16] identified as doing so. This project aims to provide evidence and insights into the factors affecting the OH behaviours of ACYP from the perspective of ACYP, their parents/carers and Dental Health Professionals (DHPs).

### 1.1 Terminology

The term ‘oral health behaviours’ here refers to behaviours to maintain OH and prevent disease [17]. Guidance recommends that children brush their teeth with a fluoride toothpaste twice a day (with the assistance of a parent/carer if required) and that consumption of sugar containing foods and drinks be minimised [17].

## 2 Methodological Approach

The research took a phenomenological approach with an emphasis on people's subjective experiences and interpretations of the world. This helps the researcher to understand what it is like to experience a specific situation or life event and enables the researcher to generate a more comprehensive and in‐depth understanding of a phenomenon [18]. This facilitated the exploration of the topic from the perspectives of the different participants and of what they perceive to be important without the constraint of a preconceived hypothesis.

## 3 Methods

Using purposive sampling, participants were recruited through local autism support organisations, social media and professional networks in Southwest England. Semi‐structured interviews were held with CYP, parents/carers and DHPs from July 2022 to January 2023. Interviews with CYP and their parents/carers were conducted online or face‐to‐face by J.E. Interviews with DHPs were held online or by telephone. Interviews followed an interview guide (one for each participation group – see Supporting Information S1.) The interview guides were developed together with members of the project PPI group and the research team. There were two interview guides for CYP, one for young children and one for older CYP. Both guides used simple accessible language to ask about caring for their teeth and their experience of going to the dentist. For older CYPs, questions were extended to include those about the strategies they have used to help with tooth brushing and their dental care history. They were piloted with autistic members of a youth club for neurodiverse CYP and their parents/carers. The DHP interview guide was piloted with independent professional colleagues.

Informed consent or assent was obtained from all study participants. The information sheets, consent and assent forms were designed for different ages and abilities and presented the information about the study and consent/assent in simple language and pictures. Only CYP who were able to understand the reason for the research and what participation involved and who were able to give informed assent or consent were included in the study. This ability was determined by the CYP's parent/carer together with the researcher. Most participants were able to easily communicate verbally with the researcher. For two of the participants, their parent's help was needed to facilitate the researchers' understanding of their communication. Only once the researcher was confident that the participant fully understood the information about the study and what taking part involved did she seek assent or consent. For children aged 5–10 years (chronological age or level of understanding commensurate with that age), the consent of their parent/legal guardian was sought for their participation in the study. For all CYPs, assent was reconfirmed verbally before the start of their interview. Written consent for participation was sought from CYP aged 11–17 who had Gillick competency [19, 20]. The decision as to whether the young person was Gillick competent was taken by the parent/guardian. For those who did not have Gillick competency consent to participate was sought from their parent or legal guardian. Written consent was required from participants aged 18–19 and all other adult participants (parents/carers, dental health professionals).

Interviews ranged in length from 25 to 70 min. The interviews were conducted by J.E., a female, Post‐doctoral Research Fellow in Public Health Dentistry with experience of, and training in qualitative research methods. She has a young autistic family member. After receiving assent/consent, J.E. met with CYP and parents/carers face‐to‐face or online to introduce herself and to help build rapport. She spoke about the reasons for the research and shared information about herself including her background, interests and hobbies. She asked about the CYPs daily lives (e.g. where they go to school, college), their hobbies, their interests, likes and dislikes and their communication preferences. This meant that, especially for the younger children, interviews could include in the preamble topics that tapped into their interests so aiding discussion. CYPs were given the choice whether or not to have their parent/carer together with them at the interview. Seven of the CYP participants chose to be interviewed individually. Two young people aged 18 were interviewed alone. Five other children who were aged between 5 and 14 years old were not interviewed together with their parents but their parents were present in the same or an adjacent room. The doors between rooms were kept open. Field notes were written after interviews. There were no repeat interviews. Transcripts were shared with participants for comments and corrections. The PPI group and participants were asked for their opinion of the findings.

The reporting of the study is guided by the COREQ checklist [21].

Ethical approval: This project was approved by the University of Plymouth, Faculty of Health Research Ethics and Integrity Committee (approval number 2022‐3029‐2777).

### 3.1 Data Analysis

Data analysis was carried out using a hybrid approach [22, 23] which combined both deductive and inductive coding. The former came from the research aims and questions. The inductive coding was open to the identification of codes reflecting important unpredicted phenomena present in the data (reflective thematic analysis [24]). An iterative approach of ongoing analysis enabled the identification of areas of interest to be explored in subsequent interviews. The deductive and inductive codes were organised into wider themes which identified patterns of meaning in the data [25]. NVivo (version 12) was used for data analysis. Coding was carried out by J.E. with S.N.

## 4 Results

Interviews were carried out with 19 ACYP (aged from 5 to 18 years), 19 parents, 1 carer and 16 DHPs This does not include two parents who dropped out because they were too busy. Details of participant characteristics are shown in Tables 1 and 2.

**Table 1:** Characteristics DHP interviewees.

DID1	Dental Hygienist
DID2	Dental Hygienist
DID3	Specialist in Paediatric Dentistry
DID5	Specialist Trainee in Special Care Dentistry
DID10	Specialist in Special Care Dentistry
DID6	GDP
DID7	GDP
DID8	Dental Nurse/OHE
DID9	Dental Nurse/OHE
DID11	Dental Nurse/OHE
D4	Community Dentist
DID12	Community Dentist
DID13	Community Dentist
DID14	GDP
DID15	GDP
DID16	Consultant in Paediatric Dentistry

**Table 2:** Characteristics CYP and parent/carer interviewees.

ID	Parent/Carer	CYP (age in years)	CYP interviewed with parent/carer present (Y/N)
ID1, ID2	Mother	Daughter (9)	Yes
ID3, ID4	Mother	Son (18)	No
ID5, ID6	Mother	Daughter (16)	Yes
ID7, ID8	Mother	Son (17)	Yes
ID9, ID10	Mother	Son (11)	No
ID32, ID33	Mother	Daughter (9)	No
ID13, ID14	Mother	Son (18)	No
ID16, ID17	Mother	Daughter (12)	Yes
ID18, ID19	Mother	Son (14)	No
ID22, ID23	Mother	Son (11)	Yes
ID24, ID25	Mother	Daughter (13)	Yes
ID26, ID31, ID37	Mother	Son (15), Son (12)	Yes
ID27, ID28, ID29, ID30	Mother, Father	Daughter (10), Son (8)	Yes
1D34, ID35, ID36	Mother, Father	Daughter (13)	Yes
ID11, ID12	Grandmother	Grandson (14)	Yes
ID21	Mother		
ID15	Mother		
ID29	Father		
ID38, ID39		Son (5), Son (8)	No

**Figure 1:**
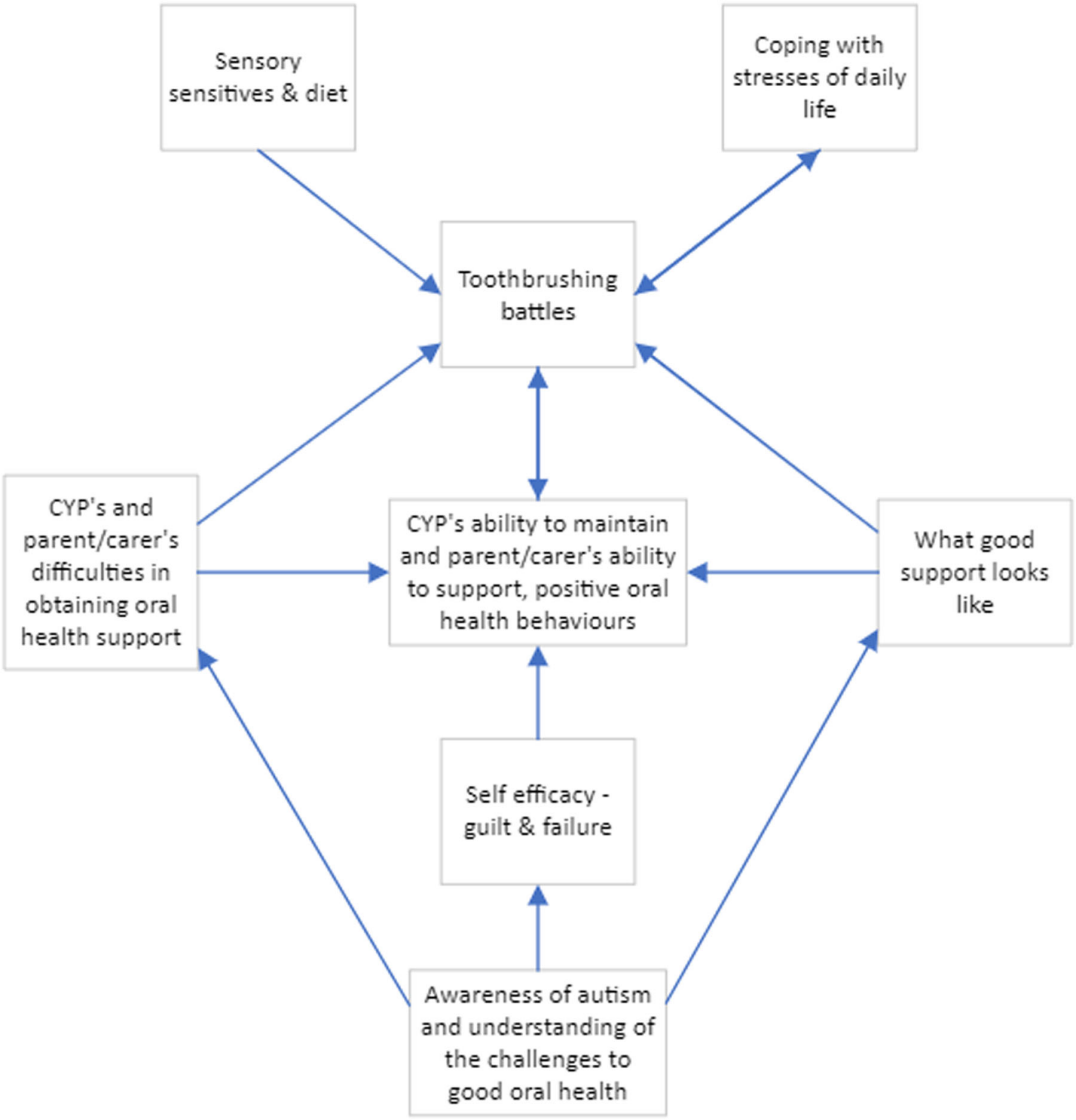
Factors influencing the oral health behaviours of autistic children and young people.

The themes identified (see Table 3 and Figure 1) are presented with illustrative quotes from the participants.

**Table 3:** Themes and subthemes.

Themes	Subthemes
Sensory sensitivities and diet	Sensory challenges to toothbrushing
	Diet choices and restrictions
Tooth brushing ‘battles’	Reluctance to brush teeth
	Maintaining physical hygiene
	Fixation with cleaning teeth
Coping with the stresses of daily life in a neurotypical world	Fitting in Masking
	Meltdowns
	At time of high stress brushing teeth is lower priority
	Stress experienced by parent/carer in supporting/advocating for child
Awareness of autism and understanding of challenges to good oral health	Lack of autism awareness in society
	Awareness among DHPs
The impact on parents of trying to care their child's oral health	Self‐efficacy
	Feelings of guilt and failure
	DHP's attitudes towards parents
CYP and parent/carers difficulties in obtaining oral health support	Lack of OH support at school
	Parents' access to advice on OH
	Peer support for parents
	Support from family and friends
What ‘good support’ looks like	Practical tips, strategies and resources
	Communicating individualised, positive and consistent advice

### 4.1 Sensory Sensitivities and Diet

CYP, parents and DHPs all cited sensory sensitivities and diet as the biggest challenges to ACYP maintaining good oral hygiene. CYP reported not liking the feel of the bristles of the toothbrush in their mouth or having ‘*something weird’* in their mouth. Whilst some were able to tolerate an electric toothbrush, others found them too noisy, ‘*whizzy’* or with ‘*too many vibrations’*. Many of the children interviewed struggled with the taste and/or feel of toothpaste. Children particularly struggled to use mint toothpaste ‘…*it's like an explosion in my mouth*!’

Some CYP had no difficulties with toothbrushing, were able to brush twice a day and were determined to do so:

ID12 (14‐year‐old boy): ‘*If I'm late getting up, the world can stop. The taxi can stay there. But I'm cleaning my teeth and that's the end of it*.’

Others were able to brush less than once a day and/or for a short period, primarily due to the challenges to brushing associated with sensory sensitivities. Some children who were assisted by their parent/carer, said that they struggled with the lack of control they felt in having someone else clean their teeth and found the physical sensation of having someone invading their personal space difficult. A number of the CYP interviewed had narrow food choices or a restricted diet and some children, under the advice of paediatric dieticians, had high‐calorie diets.

### 4.2 Tooth Brushing ‘Battles’

Parents frequently described brushing their child's teeth as a ‘battle’ mainly because their children were very reluctant to brush as they found it unpleasant or even painful. Some parents found it a battle to get their child to brush for more than a few seconds or to brush at all. This description is typical:

ID19 (Mother of 14‐year‐old boy): ‘*It's been a battle. He's not a fan of it at all. He prefers to chew a toothbrush than to actually brush. He has to be reminded to clean his teeth. And then trying to find a toothpaste that worked and that he was happy with. It's an ongoing battle really…*’

For some children, difficulty in cleaning their teeth was part of a wider issue with maintaining their physical hygiene:

ID34 (Mother of 13‐year‐old girl): ‘*It's not just brushing her teeth she can't cope with. It's sort of keeping clean in general, you know, personal hygiene, she really struggles with that*.’

CYP can become pre‐occupied with other activities such as gaming and forgo their personal care or become too overtired to clean their teeth before bed.

DHPs also highlighted that CYP can over brush:

DID1: ‘*They can be almost fixated by cleaning their teeth and cause damage and trauma with their brush or other aids that they're using.*’

### 4.3 Coping With the Stresses of Daily Life in a Neurotypical World

CYP and their parents frequently spoke about the wider experience of being autistic in a neuro‐typical world and how that impacted the CYP's ability to care for their OH. For those children attending mainstream school, the stress of having to ‘*fit in*’ and of masking their autism was exhausting. Girls, in particular, raised this as an issue.

ID25 (13‐year‐old girl): ‘*Someone like me who is a girl and skilled in the art of masking – I'm just not on people's radar… just cos I look “normal” doesn't mean to say that it's not difficult for me*.’

Both CYP and their parents spoke about the ‘*meltdowns*’ CYP experienced when they got home from school and were able to release the pressures that had built up over the day.

ID6 (16‐year‐old girl): ‘*Yeah cos when I'm at school I have to do all that stuff they tell you to do to look normal, you know like making eye contact and stuff. And it's like really hard and when I get back home sometimes I have to just go and have a lie down but Mum's nagging me about homework and that…sometimes I just lose it*.’

Given the pressures they were coping with, for some CYP looking after their teeth was not a priority and some parents felt that in the circumstances it was best not to ‘*fight*’ with the CYP to clean their teeth:

ID33 (Mother of 13‐year‐old girl): ‘*The night‐time one, if she's not… And I don't even fight her with that 'cause I know she's had a really bad –a really hard day. Like, school and – it's not easy. So, that is the last… Not the last of our worries, but it's not a priority when she's had a stressful time*.’

Parents described the stress they experienced in supporting their child to do the daily things that most families take for granted, such as getting them to school and the on‐going struggles to obtain the help and support that their child is entitled to. This was particularly difficult for those ACYP who had not yet received a formal diagnosis. It is not unusual for CYP to have to wait several years for an autism assessment leaving parents feeling in a ‘*state of limbo’*. Faced with a range of significant challenges across many aspects of their lives, for some parents, OH fell lower in their list of priorities.

The DHPs interviewed were aware of pressures on parents in supporting their child in their daily life:

DID8: *Because they* [the parents] *must be exhausted really 'cause they're having to encourage them* [their autistic child] *to do all sorts of things when they're being reluctant and not going to be complying… This poor mum was nearly in tears and when she said, ‘I don't know what we're going to do DID8,’ I said, ‘Well, we'll keep going.’*

### 4.4 Awareness of Autism and Understanding of the Challenges to Maintaining Good OH

CYP, parents and DHPs all spoke about the lack of awareness of autism in wider society. Although awareness has improved in recent years, they still felt that people had a stereotyped view of autism that did not correspond to the varied reality of how ACYP experience the world.

ID25 (13‐year‐old girl): ‘*They think that everyone with autism is like the person in Rainman*!’

Parents felt that the general lack of awareness meant that their children's behaviour was misunderstood. They often felt criticised by others, particularly where the child expressed their feelings of discomfort by behaving in ways that were seen by the public as inappropriate or ‘*naughty*’. Parents reported that a lack of awareness was sometimes apparent in the reaction of members of the dental team to the behaviour of ACYP attending the practice with negative impacts on the child's dental care experience.

ID26 (Mother of teenage boys): ‘*ID27 was really upset and sometimes to cope with that he vocalises, the receptionist was glaring at us – it was just really uncomfortable to be honest*…’

DHPs that participated in the research were aware of the challenges that ACYP can face in adopting good OH behaviours and the majority were understanding and empathetic towards them. DHPs acknowledged that not all DHPs were so well informed, and that GDPs in particular, can feel ill‐prepared to provide appropriate support of good OH behaviours in ACYP.

### 4.5 The Impact on Parents of Trying to Care for Their Child's OH

Parents spoke about the impact the toothbrushing battles had on them and their frustration that they were not able to help their children get past their sensory barriers to toothbrushing. A mother of two autistic children aged 8 and 10 described how these ‘*battles*’ made her feel:

ID27: ‘*It takes me being grumpy to push them into doing it. It makes me feel rubbish. 'Cause it's like before we've even got out the door, I've got to be the ogre and you're sort of – I don't want to be this person*.’

#### 4.5.1 Self‐Efficacy and Feelings of Guilt and Failure

Some parents expressed feelings of inadequacy and felt so overwhelmed that they could not contemplate being able to make any significant changes in their child's OH behaviour. One mother described it as ‘*just an impossible task*.’ Another expressed fatalism about the poor state of her children's teeth feeling that it was inevitable that her child would go on to have his primary teeth extracted. Others felt guilt that they had been unaware of the intensity of the sensory experience their child endured when trying to brush their teeth and were unable to help them.

The lack of professional awareness of the challenges associated with caring for ACYP's OH can lead parents to feel they are being judged or blamed for their child's poor OH:

ID34 (Mother of 13‐year‐old girl): *‘And when she was a little bit younger I felt like the dentist was telling me off. “What an awful parent you are. Look at your child's teeth.” …That annoyed me…It made me feel like a bad parent. And I wanted to say, “Look, I have done everything I can to get my child to clean their teeth properly. It is important to me. I'm not one of those parents who doesn't bother with them cleaning their teeth.” But that's what I was made to feel like.’*

Most DHPs interviewed acknowledged the feelings of guilt and failure experienced by parents and tried to reassure the parent and encourage them to continue in their efforts.

DID1: *They know that their children should be brushing twice a day. They want them to be doing more than they are and if they find out that their children have got decay …they can obviously then think, ‘Well, I should have done more,’ …. But I say, ‘Well, what has happened has happened. It's about moving forward and how we can support you…just don't beat yourself up about it.’*

Whilst nearly all DHPs interviewed were sympathetic to the challenges faced by parents, two were very critical of parents and accused them of ‘not trying’:

DID4: …*a lot of the time parents are quite defensive, coming to you in an attitude already and then everything you say, ‘Well, we need to cut a little bit on the sugar if possible,’ ‘No, he's autistic. He has to have it. I can't say no to him.’ … It's like almost hanging all the blame on autism and it's like, parents, you know, don't want to do their parental job…Some of the parents, they blame everything on autism by not looking after the child properly.*

DHPs recognised that CYP and children can receive conflicting advice from health professionals, particularly around diet. This can contribute to uncertainty and lead parents to feel that they are being ‘*judged*’ or ‘*blamed*’ for their child's poor OH:

ID17: (Mother of 12‐year‐old girl): ‘*So, from a paediatrician request, we go for a high‐calorie diet. I've said from day dot, “This is going to impact her teeth.” … And that surprise, surprise, that has impacted on her teeth. But I think you're made to feel – as a parent – bad* [by DHP]. *And it's not like I want her to eat all this chocolate because I don't but because her appetite is so poor and that's what we're told to go with.*’

### 4.6 Children and Parents/Carers' Difficulties in Obtaining OH Support

Some CYP who attended mainstream school commented that they would not want to seek support from anyone there as it would highlight their difference:

ID6 (16‐year‐old girl): ‘*Yeah, I mean if I had to talk to someone else* [other than her Mum or dentist] *they wouldn't get it, they would just think I'm super weird.*’

Another mother of a 13‐year‐old girl reported that there was very little support and understanding in mainstream education for neuro‐divergent children and her daughter would be too embarrassed to speak about her struggles with OH with anyone there.

Young people felt that it was just expected that by the time they reached secondary school age they wouldn't have any problems cleaning their teeth:

ID18 (14‐year‐old boy): ‘*No, nothing in secondary school, I don't think. 'Cause they expect you to already know how to brush your teeth properly and all that…They don't understand that you can know how to do it but not be able to do it*’

Parents described how they struggled to find out how they could help their child to overcome the challenges they experienced in maintaining their OH. Parents felt alone and often did not realise other parents were also struggling with these issues. They felt that OH was not prioritised by those organisations and services tasked with supporting autistic children and their families. They experienced a lack of support in caring for their child's teeth pre and post autism diagnosis which presented a lost opportunity to avoid the establishment of ‘*bad habits*’ and dental decay:

ID19: (Mother of 14‐year‐old boy): ‘*When we had the diagnosis, we were basically given this life‐altering diagnosis and “Off you go. There you go. There's your diagnosis.” “Okay, great. What do I do from here?” There's literally nothing. There is no help. Including dental help. There is none. So, you make it up as you go along.*’

Finding local support groups and speaking to other parents helped parents feel less isolated and served to fill the gap left by the lack of support from ‘*official*’ sources. One parent in reaction to the lack of support, created a social media community where parents could exchange experiences and strategies to help them care for their child's teeth:

ID2 (Mother of 17‐year‐old boy): *I set up a local Facebook page 'Cause I found there was no help out there. So, I thought ‘Well, if can attract other autistic parents and we just share information. That helps. And so the family of autistic people – once you link in with them – can be a really helpful network. Because we understand the struggles and we understand the fights and the battles. And sometimes when we learn things, we're keen to get out there and tell other people about it so they can access it too. So, that's probably the best self‐help network.’*

Participants reported that families play an important role in helping ACYP care for their OH. Examples cited included siblings and other family members modelling good OH behaviours, providing encouragement and celebrating the child's achievements. The behaviour of family members can, however, be unintentionally detrimental to the child's OH. Some parents mentioned how their considerable efforts to control their child's intake of sugary foods were undermined by grandparents and other family who wanted to ‘*treat*’ the child by giving them sweets, cakes and sugary drinks. Parents recognised that this came from not wanting to ‘*deprive*’ the child and not wanting to treat them differently to other children. This was difficult and often frustrating for parents and added to their battles.

### 4.7 What ‘Good Support’ Looks Like

CYP, parents and DHPs were asked how ACYP and their parents could be best supported to care for their OH. In general, the CYP felt that they got the support they needed from their parents/carers, one of the older CYP suggested that parents/carers be given the skills to help provide that support. Both parents and DHPs emphasised the need for practical support to meet the individual needs of ACYP and their parents/carers.

#### 4.7.1 Practical Tips, Strategies and Resources

CYP reported receiving support to care for their teeth from their parents, DHPs and, for some, from school. Where there was a toothbrushing programme at school, establishing routines and brushing with their peers had a positive impact on CYP's toothbrushing at home. For some children, it was not until they started toothbrushing at school that they started toothbrushing at home.

Two children spoke about how learning the steps of toothbrushing at school using the Picture Exchange Communications System (PECS) helped:

ID01 (10‐year‐old girl): ‘*I like the PECs…Step one. Put – get a toothbrush. Step two. Get toothpaste. Put it on the toothbrush. Step three. Go the sink. Step…*’

Parents wanted someone with whom they could talk through their problems and who could give them practical tips, advice and strategies on how to brush their child's teeth. Both parents and DHPs said that making OH information and resources more widely available in the community would help parents/carers to support their children to maintain their OH. This included access to affordable resources such as frui‐flavoured, flavourless or low‐foaming toothpastes and specialist toothbrushes. Other suggestions included delivering OH training to parents of ACYP in schools, youth clubs and playgroups; including OH information and support in resources supplied by services such as Child Development Centres; having OH educators in the community to advise ACYP and their parents on brushing/OH and training parents as peer educators.

ID17 (Mother of children aged 4 and 12): ‘– *it would be good if dentists could send you stuff out like timetables and fun pictures and step‐by‐step guides because a lot of autistic children don't listen to parents because they feel we are bossing them. But when it comes from other people, they then actually listen a little bit more*.’

Table 4 lists the strategies that CYP, parents/carers and DHPs found useful in establishing positive OH behaviours.

**Table 4:** Strategies used by CYPs, parents/carers and DHP to support autistic CYP to maintain oral hygiene.

CYP strategies	Parental strategies	DHP strategies
Working around daily routine of going to school Breaking toothbrushing down into steps Brushing with siblings	Trying products – trying different types of toothpaste and toothbrush that the child can tolerate Modelling – using themselves or teddy bears/dolls to model tooth brushing and to demonstrate brushing techniques. Encouraging longer brushing using apps on their phone or tablets. Rewards – using rewards to motivate their child to brush their teeth, for example, star charts and giving the child time on the play station or with their favourite game. Breaking down the process – breaking tooth brushing into a number of steps.	Using props – using models to demonstrate to the child how to clean their teeth. Encouraging CYP and parents to brush at a time that best suits the child and family routine. Introducing sensations – encouraging children to play with water, and feeling toothpaste and toothbrushes with their fingers. Rewards – Giving praise and stickers Encouraging family members to motivate – instructing parents and siblings in toothbrushing techniques and encouraging them to model good techniques Continual reinforcement – seeing the child and their parent over a number of appointments, gradually building up advice by delivering in ‘nuggets’.

#### 4.7.2 Communicating Individualised, Positive and Consistent Advice

CYPs and parents said that they wanted advice that related to them and their particular circumstances and not just generic messages. They wanted this to be communicated in a way that meets the needs of the child. One young person summed up a key principle of communication with ACYP:

ID12 (14‐year‐old boy): ‘*I mean, autism and ADHD and all that stuff is such a spectrum. I mean, my case, I usually like quite chatty people… But I know some people are the exact opposite where they don't want to hear any talking, they just want it to get it over with. So, it is not,* “*This is what would be best for autistic people.” Ask* “*What would be best for you?*”*’*

The mother of a 10‐year‐old girl commented on the importance of the advice that is given by DHPs being relevant to the particular needs of the child:

ID02: *The dentist told us to use an electric toothbrush because they are ‘most efficient’, that maybe true but no use being ‘efficient’ if it's too noisy for the child to ever use*!

DHPs, especially those working in community or specialist dental services emphasised the need to provide individualised advice that is realistic and relevant for the context of the lives of the child and their family:

DID1: ‘…*I ask them what their current routines are and then try and tailor it in and around what they already do. So, if they've got younger children, what I would do is if it's pre‐school children is try and get them to get their older children to school first…*’

By speaking directly to the child and finding out about their routines and how they spend their days, they can pinpoint opportunities in the day for the child to brush their teeth:

DID1: *So, for example, if someone is gaming, I will say,* ‘ *Right, well when you've lost a life or someone that you're playing with online isn't around or you need to go to the loo, that's the time to do it.*’

DHPs highlighted the importance of focussing on progress, not on achieving the ‘*ideal*’ and of offering alternative ways of doing things.

DID7: *…I say to parents,* ‘ *Look, one good brush at the end of the day is better than two rubbish brushes. So, kind of pick your battles and do it at bedtime… Even if it's not necessarily perfect, it's got some value and it will help…that's the important thing and the parents knowing that what they're doing is valuable and just to keep going*.’

DHPs also emphasised the importance of the timing of advice, the need for encouragement, positivity and the celebration of achievements, however small and of avoiding escalating difficult situations.

## 5 Discussion

### 5.1 Main Findings

This research provides insight into the factors that influence ACYP's OH behaviours and how the OH of ACYP can best be supported. What emerges strongly is the extent to which the wider environment impacts the OH behaviours of ACYP. Many ACYP face significant challenges to maintaining good OH, parents/carers feel alone in their efforts to support their child to overcome these challenges, and DHPs can find it difficult to provide the support that is needed.

Living in a neurotypical world can be difficult for ACYP. It can be exhausting to read the social cues and make the effort to communicate [26], leaving little energy to struggle with things like toothbrushing at the end of the day. This highlights the significant stress many CYPs experience during a school day, particularly those who feel under pressure to mask and the impact this can have on their mental health [27]. CYP can feel that they are alone in having difficulties with maintaining their OH accentuating their feelings of ‘*difference*’.

This study highlights the levels of stress experienced in daily life by the parent/carers of ACYP. This can be a barrier to maintaining children's OH [28]. Parents of autistic children report high levels of demand in their parental role [29] and higher levels of stress than parents of neurotypical children [30] which can cause fatigue and be detrimental to their mental health and well‐being [31]. In our interviews, it was clear that parents wanted the best for their child, including good OH but, as the key figures in supporting and advocating for their child in all aspects of their life, they were often overwhelmed with demands not experienced by parents of neurotypical children. The stress and exhaustion they experienced meant that for some the battles with toothbrushing were just one battle too many. Moreover, although parents wanted to brush their child's teeth regularly and to control their child's consumption of sugary foods and drinks, some parents had little belief in their ability to implement this behaviour and achieve their goal. This has important implications for DHPs practice as it necessitates the need to support motivation and health behaviour change of the parent and the CYP [32].

Parents described experiencing direct verbal criticism and felt criticism [33] from DHPs in regard to their inability to ‘*make*’ their child brush their teeth and to restrict sugar in their diet. Parents also experienced criticism from the wider public for their autistic child's behaviour. This exemplifies the parent‐blame discourse that exists in practice across all domains [34]. There are strong social norms around ‘*good*’ parenting and the role of the mother in ensuring their child's health and well‐being and ability to follow social norms within the social sphere [35]. The parents' ‘*failure*’ to comply with these norms engenders a range of emotions including shame, guilt and anxiety and can impact health care seeking and self‐efficacy [36, 37]. There needs to be a suspension of judgement by DHPs otherwise this will act as barrier to engagement with OH services by CYP and their parents and may negatively impact parents perceived ability to develop positive OH behaviours.

CYPs emphasised the importance of communicating how to brush their teeth in a way that is accessible to them. Those CYPs who attended specialist schools were offered OH education. They felt that being giving specific itemised instructions using familiar communication tools was particularly helpful in establishing positive OH behaviours. Embedding toothbrushing into the structured school day helped facilitate toothbrushing routines at home. DHPs who were more experienced in working with ACYP were able to support and build on these first steps to help overcome the challenges the CYP faced. Other DHPs were aware of the challenges but didn't always feel equipped to provide appropriate support, especially given what they perceived to be a lack of training and appropriate communication resources.

### 5.2 Implications for Practice and/or Services

#### 5.2.1 Training and Resources for Parents, Family and Professionals

Focusing on improving parent's skills in ways that are supportive, child‐specific and realistic in the context of their lives may help reduce stress, anxiety and guilt and increase parents' feelings of self‐efficacy [28, 38, 39]. Parenting self‐efficacy is associated with lower levels of stress, anxiety and guilt in parents with autistic children [39] and is an important factor in parents' control over their child's toothbrushing and diet [38]. Research indicates the potential of parent‐led interventions to improve OH behaviours in CYP [40, 41].

Training and resources are needed to raise the priority of OH for all health and social care professionals and to enable them to deliver clear and consistent OH messages that relate to the needs of the autistic child [41, 42]. Greater understanding and training around sensory issues and adjustment of practice is needed for dentists. The development of suitable resources to share with the wider family may help support a more united approach to OH [43]. Toothpaste and dental products that support sensorial differences should be available on prescription or at the same price as other dental products.

#### 5.2.2 Providing Advice and Signposting

Research indicates UK parents can receive inconsistent and conflicting OH advice from child health professionals in primary care [44]. Health professionals need to communicate with one another and explain clearly to the CYP and their parents/carers how and why they have taken the decisions they have and how they may impact OH. It is the responsibility of the professionals to help parents navigate areas of uncertainty. Better communication about OH between the various organisations that support ACYP and their families and improved interprofessional working is needed. Providing clarity about how and where to signpost them for help with OH has the potential to improve the situation for struggling families.

#### 5.2.3 Creating a Positive OH Environment

Improved OH for ACYP needs to be built on a foundation of public health approaches which give greater priority to OH. The establishment of a wider community‐based system of oral support for ACYP and parents across professional boundaries would be of great benefit [45]. School toothbrushing programmes have been shown to improve access to OH support for young autistic children [46] and should be made more widely available. OH support should be continued and consolidated in secondary school.

#### 5.2.4 Raising Awareness of Autism

There is a need for greater awareness of the particular challenges that ACYP can experience with maintaining their OH. In the past decade, there has been a significant increase in awareness of autism and neurodivergence [47]. There is still more work to be done in terms of educating people on how ACYP may experience the world, on communication through behaviour and improving the reciprocity of communication. Greater reciprocity will support respect for difference and help prevent unconscious bias and stereotyping, which can benefit all in society. Tackling this important barrier to healthcare access is crucial [48].

### 5.3 Strengths and Limitations

The voice of the ACYP is absent from previous OH studies [10]. The main strength of this research is that it has explored factors influencing OH behaviours through the perspective of ACYP, their parents/carers and DHPs. This allows for greater insights into the complex range of factors that influence the OH behaviours ACYP.

Five of the sixteen DHPs interviewed had autistic family members and most of the DHPs interviewed were specialists or community DHPs. It may be that the DHPs who were interested in, and chose to take part in this research, were those who had a greater awareness of the challenges autistic children and their families face in maintaining good OH and were more empathic towards them. Whilst the DHP were recruited from across the southwest of England the CYP and parents were residents in one city in the region. The experiences of ACYP and families, in particular the support available to them, may differ by location.

### 5.4 Further Research

Areas for future research include identifying how training for parents can best be delivered; mapping of the OH support and services available to ACYP and their families across England; identifying the key elements of a positive community OH environment and how they might be established.

## 6 Conclusions

The goal of reducing OH inequalities for autistic children and adults requires a commitment to change. Providing early support, strategies and resources to CYP and their parents/carers has the potential to help the establishment of positive OH behaviours. This needs to be implemented along with the creation of a system where health, social care and other professionals are aware of the challenges faced by ACYP, can provide informed, consistent OH advice and where relevant organisations such as schools are enabled and financed to support OH for all CYP.

## Author Contributions

**Jo Erwin:** investigation, writing–original draft, methodology, writing–review and editing, formal analysis. **Sarah Neill:** conceptualization, funding acquisition, writing–review and editing, methodology, formal analysis, supervision. **Tara Vassallo:** writing–review and editing, methodology, investigation. **Isaac Vassallo:** investigation, writing–review and editing, methodology. **Rob Witton:** conceptualization, funding acquisition, writing–review and editing, methodology, supervision. **Martha Paisi:** conceptualization, funding acquisition, writing–review and editing, methodology, formal analysis, project administration, supervision.

## Ethics Statement

Ethical approval for this study was given by the University of Plymouth Research and Integrity Committee (#3029).

## Conflicts of Interest

The authors declare no conflicts of interest.

## Supporting information

Supporting information.

## Data Availability

The anonymised data that support the findings of this study are available from the corresponding author [M.P.] upon reasonable request.
