# Supplementary material for: Factors Influencing the Oral Health Behaviours of Autistic Children and Young People: A Qualitative Study
Source: Health Expect. 2024 Dec 17;27(6):e70130. doi: 10.1111/hex.70130 (PMC11652779; doi:10.1111/hex.70130)
Supplement: Supplementary file 1 — Supporting information. [file HEX-27-e70130-s001.docx]

**Supplement one**

**Interview guides**

**Interview guide - Parents/Carers**

Prompts in [ ]

INTRO

Tell me about (child’s name) [when diagnosed with autism, his/her characteristics]

ORAL CARE

Let’s talk about toothbrushing -

What has been your experience looking after (name)’s teeth at home?

What difficulties have you had?

What has helped? [things came up with, things learnt from others]

DENTAL CARE

Ok thanks, let’s talk about going to the dentist –

Has your child ever been to the dentist?

**If yes**

How old were they when they first went to the dentist. How was that first visit?

How have visits to the dentist been since then? [positive and negative experiences]

Please tell me a little about the dentists you’ve seen [type of dentist, characteristics/qualities of dentist and wider dental team, positive and negative experiences]

What would make going to the dentist easier for you and your child?

**If no**

Please tell me a little about why s/he hasn’t been to the dentist

What would make going to the dentist easier for you and your child? [Changes would like to see]

**Interview guide – young people and older children**

Prompts in [ ]

INTRO

Tell me about what you did to get ready for school/college [refer to the days activity] today? [things they do every day]

ORAL HEALTH CARE

Let’s talk about looking after your teeth -

What do you do to look after your teeth? [toothbrushing, flossing, mouth wash etc]

Why do you do that?

Do you like brushing your teeth? [why]

What is the most difficult thing about looking after your teeth?

What has helped? [things came up with, things learnt from others]

DENTAL CARE

Ok thanks, let’s talk about going to the dentist –

Have you ever been to the dentist?

**If yes**

How old were they when you first went to the dentist.

If you can remember that first visit, how was it?

How have visits to the dentist been since then? [positive & negative experiences]

What would make it easier for you to go to the dentist?

[What would make going to the dentist a nicer experience for you?]

**If no**

Please tell me a little about why you haven’t been to the dentist

What would make going to the dentist easier for you? [What changes would like to see]

**Interview guide – young children**

Prompts in [ ]

INTRO

Tell me about what you did to get ready for school [refer to the days activity] today? [Things they do every day]

ORAL HEALTH CARE

Let’s talk about looking after your teeth -

What do you do to look after your teeth? [toothbrushing, flossing, mouth wash etc]

Do you like brushing your teeth? [why]

What is hardest thing about looking after your teeth?

What makes it easier for you to look after your teeth?

DENTAL CARE

Ok thanks, let’s talk about going to the dentist –

Have you ever been to the dentist?

**If yes**

Do you like going to the dentist? [why]

What would make going to the dentist better? [what would you change at the dentists]

**If no**

Do you know why you haven’t been to the dentist?

If yes – why haven’t you been?

What would make going to the dentist easier for you?

**Interview guide - dental professionals**

Prompts in [ ]

INTRO

What is your role?

How long have been working in this role?

EXPERIENCE WITH AUTISTIC CHILDREN AND ADOLESCENTS:

Tell me a little about your experience providing care to autistic children and autistic teenagers

Explore –

How much experience

Nature of experience

ORAL HEALTH CARE

Thinking about maintaining oral health at home and establishing good oral health behaviour -

What are the challenges that the autistic children and teenagers that you meet have with oral health care?

How can they best be supported to look after their oral health?

What are the challenges to providing that support?

How can those challenges be overcome?

PROVIDING/ACCESSING DENTAL CARE

Thinking about the service you provide in the dental clinic -

What are the challenges that the autistic children and teenagers that you meet have with coming to the dentist?

How can these challenges best be overcome?

What are the difficulties in overcoming them?

What have you found works well?

How confident do you feel in providing dental care to autistic children and teenagers? [why is that]

What do you think would help you and your practice to better serve autistic children and teenagers?
